# A multiple-center clinical evaluation of a new real-time reverse transcriptase PCR diagnostic kit for SARS-CoV-2

**DOI:** 10.2217/fvl-2020-0299

**Published:** 2020-10-28

**Authors:** Jing-Jing Guo, Yan-Hua Yu, Xue-Ying Ma, Ya-Nan Liu, Qian Fang, Pei Qu, Jie Guo, Jin-Li Lou, Ya-Jie Wang

**Affiliations:** 1 ^1^Department of Clinical Laboratory, Beijing Ditan Hospital, Capital Medical University, Beijing, China; 2 ^2^Department of Clinical Laboratory, Beijing Youan Hospital, Capital Medical University, Beijing, China

**Keywords:** detection, evaluation, real-time PCR Kit, SARS-CoV-2

## Abstract

**Aim:** The outbreak of severe acute respiratory syndrome-coronavirus 2 (SARS-CoV-2) has had serious repercussions worldwide. This study was aimed to evaluate the accuracy of a new kit for detection of SARS-CoV-2 compared with similar detection kit. **Materials & methods:** A total of 500 subjects were included and tested with both the new test and control kits. Clinical diagnosis results were taken as the reference standard. **Results:** Compared with clinical diagnosis, the sensitivity of the test kit was 82.64%, specificity was 98.45% and total coincidence rate was 90.80%. The total coincidence rate, sensitivity and specificity between control kit and clinical diagnosis were 89.20%, 78.10% and 99.61%, respectively. **Conclusions:** The new kit was comparable to the similar detection kit for detection of SARS-CoV-2 in sensitivity, specificity and total coincidence rate.

In December 2019, a novel severe acute respiratory syndrome-coronavirus 2 (SARS-CoV-2) caused a disease outbreak [1]. The SARS-CoV-2 as a global pandemic has affected 212 countries with total confirmed cases 5,575,762 and 349,302 deaths as of May 26, 2020. The main clinical symptoms of SARS-CoV-2 patients are fever, cough, chest discomfort/tightness and fatigue; a small number of patients have had gastrointestinal symptoms [2,3]. Elderly adults and people with underlying chronic diseases are more susceptible to infection and prone to more severe cases [4]. At present, there is no specific antiviral drug or therapeutic vaccine for SARS-CoV-2. The key to prevent and control SARS-CoV-2 lies in the early detection and isolation of patients [5]. Therefore, accurate and rapid diagnosis of SARS-CoV-2 infection is extremely important to disrupt the transmission chain.

Recognizing the urgency and necessity of rapid diagnosis, the China Center for Drug Evaluation announced a ‘green channel’ in January 2020 to review and approve the SARS-CoV-2 testing kits and other biomedical interventions. Real-time reverse transcription PCR (RT-PCR) is the gold-standard for SARS-CoV-2 detection with high sensitivity and specificity [6,7]. Eight RT-PCR kits for detection of SARS-CoV-2 have been approved in China [8]. A new commercial multiple real-time PCR kit for detection of SARS-CoV-2 was developed by Beijing Applied Biological Technologies Co. In this study, we aimed to evaluate the accuracy of the new kit for detection of SARS-CoV-2. We selected a kit with the same methodology, applicable sample type and intended use, produced by Shanghai ZJ Bio-Tech Co. Ltd as the control. Here we report a multicenter clinical evaluation of a new kit for detection of SARS-CoV-2 using a large number of clinical specimens.

## Materials & methods

### Study design & clinical samples

The objective of the study was to evaluate the accuracy of the Multiple Real-Time PCR Kit (hereafter, ‘test kit’) for the detection of SARS-CoV-2 developed by Beijing Applied Biological Technologies Co. compared with a similar detection kit (control kit, Shanghai ZJ Bio-Tech Co. Ltd, Shanghai, China). Specimens were collected between February 14 and February 18, 2020, and were held at 4°C if all testing could be completed within 24 h. 500 subjects (395 oropharyngeal swab specimens; 167 sputum specimens) with suspected or clinical diagnosis of SARS-CoV-2 or with history of close contact from three centers in China were enrolled in this study. Among these subjects, 62 underwent double sampling. Patients with fever, cough, chest discomfort/tightness and other clinical symptoms were also included in the study. The results of clinical diagnosis were taken as the reference standard. The clinical diagnosis of SARS-CoV-2 infection was according to the latest guideline of Diagnosis and Treatment of Pneumonitis Caused by SARS-CoV-2 (trial seventh version) published by the Chinse government [9]. Diagnosis was based on clinical history, laboratory, chest radiographic findings and nucleic acid-based assays. The remaining specimens for clinical testing were collected and tested with two RT-PCR diagnostic kits. This study was conducted in accordance with Declaration of Helsinki and Good Clinical Practice Guidelines and approved by the ethics committee institutional review board of each participating center.

### Performance verification of the test kit

The performance verification of the test kit was conducted according to the Measures for the Administration of Registration of In-Vitro Diagnostic Reagents, including lowest detection limit, freeze–thaw stability, cross-reactivity and anti-interference ability. The lowest detection limit for the test kit was determined as the lowest concentration with 90–95% of the tested samples in positive. The three batches of test kits were frozen (–20 ± 5°C) and thawed 3, 6, 9, 10 or 11 times at 25 ± 2°C, respectively, to verify the freeze–thaw stability. To evaluate potential cross-reactivity, 56 pathogens with the same infection site or similar symptoms as SARS-CoV-2 were detected. These organisms included human coronavirus (229E, OC43, HKU1 and NL63), severe acute respiratory syndrome-related coronavirus (SARSr-CoV), seasonal influenza A (H1N1, H3N2, H5N1 and H7N9) viruses and adenovirus (type 1, 2, 3, 4, 5, 7, and 55), among others. Anti-interference ability was measured in the presence of various interfering substances. For this purpose, two independent oropharyngeal swabs and sputum specimens were spiked with potential interfering substances that could be collected with the sample. Interfering substances included 2.5%, 5.0% and 7.5% blood (v/v), mucoprotein (0.45, 0.9 or 1.35 mg/ml), beclomethasone (2.5 mg/ml), dexamethasone (5 μg/ml), phenylephrine (50%, v/v) and oxymetazoline (50%, v/v), for example.

### Real-time PCR assay for screening of SARS-CoV-2

RNA was extracted from different specimens according to the manufacturer's instructions. Both RT-PCR kits targeted the *ORF1ab* and nucleoprotein gene regions. If two targets tested positive, the subject was considered to be laboratory confirmed. For the test kit developed by Beijing Applied Biological Technologies Co., a cycle threshold value (Ct-value) of 38 or less was considered a positive test, whereas a Ct-value greater than 40 was defined as a negative test. Specimens with a Ct-value of 38–40 need to be retested. If the repeated Ct-value was less than 40 and an obvious peak was observed, the retest was considered as positive. For the control kit developed by Shanghai ZJ Bio-Tech Co., a Ct-value less than 37 was defined as a positive test, whereas a Ct-value of 40 or more was considered as a negative test. Specimens with a Ct-value of 37–40 need to be retested. If the repeated Ct-value was less than 40 and an obvious peak was observed, the retest was considered positive. When both sputum and oropharyngeal swab specimens were collected from the same subject, sputum specimen was included in the final statistic.

### Statistical analysis

All statistical analyses were performed by using SPSS version 22.0 (SPSS Institute, Chicago, IL, USA). Quantitative data were expressed as means ± SD. Qualitative data were expressed as number and percentage. Sensitivity (positive coincidence rate), specificity (negative coincidence rate), total coincidence rate, positive predictive value (PPV), negative predictive value (NPV) and Kappa index were used for degree of agreement of the two kits. Statistical significance was set at p < 0.05.

## Results

### Performance verification of the test kit

As shown in Table 1, the positive rates of the three batches of kits were all 100% when the sample concentration were 1.0 × 10^3^, 5.0 × 10^2^ and 2.0 × 10^2^, respectively. Therefore, the lowest detection limit was 2.0 × 10^2^ copies/ml. The results obtained after 11 freeze–thaw cycles of the test kit showed no significant difference between the results obtained after 0 freeze–thaw cycles (Table 2). In addition, there was no cross-reaction with other pathogens, and no interference was observed with the interfering substances.

**Table 1.** Validation of the lowest detection limit.

Concentration (copies/ml)	*ORF1ab*			N			E		
	Batches 1	Batches 2	Batches 3	Batches 1	Batches 2	Batches 3	Batches 1	Batches 2	Batches 3
1.0 × 10^3^	100%	100%	100%	100%	100%	100%	100%	100%	100%
5.0 × 10^2^	100%	100%	100%	100%	100%	100%	100%	100%	100%
2.0 × 10^2^	100%	100%	100%	100%	100%	100%	100%	100%	100%
1.0 × 10^2^	80%	95%	90%	80%	90%	90%	80%	90%	90%

Target gene: E: Envelope; N: Nucleoprotein; *ORF1ab*.

**Table 2.** Validation of the freeze–thaw stability.

Freeze–thaw cycles			0	3	6	9	10	11
Negative (N1–N10)			10/10	10/10	10/10	10/10	10/10	10/10
Positive (P1–P2)		*ORF1ab*	2/2	2/2	2/2	2/2	2/2	2/2
		N	2/2	2/2	2/2	2/2	2/2	2/2
		E	2/2	2/2	2/2	2/2	2/2	2/2
L1		*ORF1ab*	+	+	+	+	+	+
		N	+	+	+	+	+	+
		E	+	+	+	+	+	+
L1–10		*ORF1ab*	+	+	+	+	+	+
		N	+	+	+	+	+	+
		E	+	+	+	+	+	+
L1–100		*ORF1ab*	-	-	-	-	-	-
		N	-	-	-	-	-	-
		E	-	-	-	-	-	-
J1	*ORF1ab*	Positive	10/10	10/10	10/10	10/10	10/10	10/10
		CV (%)	0.42	0.37	0.41	0.40	0.38	0.42
	N	Positive	10/10	10/10	10/10	10/10	10/10	10/10
		CV (%)	0.25	0.24	0.36	0.21	0.36	0.24
	E	Positive	10/10	10/10	10/10	10/10	10/10	10/10
		CV (%)	0.42	0.39	0.41	0.41	0.39	0.42
J2	*ORF1ab*	Positive	10/10	10/10	10/10	10/10	10/10	10/10
		CV (%)	0.35	0.35	0.32	0.31	0.31	0.32
	N	Positive	10/10	10/10	10/10	10/10	10/10	10/10
		CV (%)	0.41	0.47	0.32	0.31	0.32	0.46
	E	Positive	10/10	10/10	10/10	10/10	10/10	10/10
		CV (%)	0.72	0.63	0.70	0.69	0.66	0.72

CV: Coefficient of variation; E: Envelope; J1 and J2: Precision reference; L1: Lowest detection limit reference; N: Nucleoprotein; N1–N10: Negative reference; P1–P2: Positive reference.

### Baseline characteristics

A total of 500 subjects (258 males, 242 females) were enrolled in this study from February 14 to February 18, 2020. The baseline characteristics of included subjects are shown in Table 3. A total of 562 specimens were collected, including 395 oropharyngeal swabs specimens and 167 sputum specimens. A total of 62 subjects underwent double sampling. The age range of subjects was 0.75 (9 months)–93 years old. The majority of the subjects were aged 20–80 years, of which 40.8% subjects aged 20–40 years, 30.6% subjects aged 40–60 years, and 16.8% subjects aged 60–80 years. Among the 500 subjects, 94 had symptoms. The main symptoms of included subjects were fever (9.0%), pneumonia (2.6%), fatigue (2.0%), pulmonary infection (1.6%), cough (1.4%) and chest discomfort/tightness (1.4%).

**Table 3.** Baseline characteristics of included subjects.

Characteristic	n (%)
Gender, male/famale	258 (51.6)/242 (48.4)
Specimens (n = 562)	
Oropharyngeal swabs	395 (70.3)
Sputum	167 (29.7)
No. of double specimens	62 (12.4)
Age (years), range	0.75–93
Age groups (years)	
<20	32 (6.4)
20–40	204 (40.8)
40–60	153 (30.6)
60–80	84 (16.8)
>80	27 (5.4)
Symptoms	
Fever	45 (9.0)
Cough	7 (1.4)
Chest discomfort/tightness	7 (1.4)
Fatigue	10 (2.0)
Pneumonia	13 (2.6)
Pulmonary infection	8 (1.6)
Bronchial inflammation	4 (0.8)

### Comparison of two commercial RT-PCR diagnostic kits

All subjects were detected by the two kits, and were diagnosed clinically according to the latest guideline of Diagnosis and Treatment of Pneumonitis Caused by SARS-CoV-2 (trial seventh version). For test kit, 22 specimens with a Ct-value of 38–40 needed to be retested. For control kit, 12 specimens with a Ct-value of 37–40 needed to be retested. Among the 500 subjects, test kit results of 454 subjects were consistent with clinical diagnosis (200 were positive, 254 were negative), and 46 subjects were inconsistent (Table 4). Compared with clinical diagnosis, the sensitivity of test kit was 82.64% (95% CI: 77.27–87.20), the specificity was 98.45% (95% CI: 96.08–99.58) and the total coincidence rate was 90.80% (95% CI: 87.92–93.19). The PPV was 98.04% (95% CI: 96.14–99.94), and the NPV was 85.81% (95% CI: 79.49–89.78).

**Table 4.** The results of 500 subjects detected by two kits and clinical diagnosis.

		Clinical diagnosis		Total	Sensitivity (%) (95% CI)	Specificity (%) (95% CI)	Total coincidence rate	PPV (%)	NPV (%)	Control kit		Total	Total coincidence rate	Kappa index
		Positive	Negative							Positive	Negative			
Test kit	Positive	200	4	204	82.64 (77.27–87.20)	98.45 (96.08–99.58)	90.80 (87.92–93.19)	98.04 (96.14–99.94)	85.81 (79.49–89.78)	215	18	233	96.44 (94.56–97.81)	0.9259
	Negative	42	254	296						2	327	329		
	Total	242	258	500						217	345	562		
Control kit	Positive	189	1	190	78.10 (72.35–83.14)	99.61 (97.86–99.99)	89.20 (81.14–91.78)	99.47 (99.46–1.00)	82.90 (82.86–87.09)					
	Negative	53	257	310										
	Total	242	258	500										

Calculations: NPV = true negative/(true negative + false negative); PPV = true positive/(true positive + false positive); Sensitivity = true positive/(true positive + false negative); Specificity = true negative/(true negative + false positive); Total coincidence rate = (true positive + true negative)/total number.

NPV: Negative predictive value; PPV: Positive predictive value.

Among the 500 subjects, control kit results of 446 subjects were consistent with clinical diagnosis (189 were positive, 257 were negative), and 54 subjects were inconsistent (Table 4). The total coincidence rate between control kit and clinical diagnosis was 89.20% (95% CI: 86.14–91.78), with a sensitivity of 78.10% (95% CI: 72.35–83.14), a specificity of 99.61% (95% CI: 97.86–99.99), a PPV of 99.47% (95% CI: 99.46 to 1.00), and a NPV of 82.90% (95% CI: 82.86 to 87.09).

As shown in Table 4, test kit results of 542 specimens were consistent with control kit (215 were positive, 327 were negative) and 20 specimens were inconsistent. The total coincidence rate between two kits was 96.44% (95% CI: 94.56 to 97.81), and the Kappa index was 0.9259 (p < 0.05), indicating the accuracy of test kit for detection of SARS-CoV-2 was comparable to control kit. Among the 20 inconsistent specimens, 18 were positive and 2 were negative by test kit. In addition, the experimental kit results of 15 specimens were consistent with clinical diagnosis (14 were positive, 1 was negative).

### Subgroup analysis by age

The results of subgroup analysis by age are shown in Table 5. Among the 389 subjects aged ≤60 years, test kit results of 362 subjects were consistent with clinical diagnosis. Compared with clinical diagnosis, the total coincidence rate of test kit was 93.06% (95% CI: 90.46 to 95.58), the sensitivity was 84.70% (95% CI: 65.84–1.00), the specificity was 99.54% (95% CI: 97.36–99.99), the PPV was 99.31% (95% CI: 96.22–99.98) and the NPV was 89.34% (95% CI: 85.13–93.22). The total coincidence rate between test kit and control kit was 96.66% (95% CI: 94.35–98.21) and the Kappa index was 0.9274.

**Table 5.** The results of subgroup analysis by age.

Test kit		Clinical diagnosis		Total	Sensitivity (%) (95% CI)	Specificity (%) (95% CI)	Total coincidence rate	PPV (%)	NPV (%)	Control kit		Total	Total coincidence rate	Kappa index
		Positive	Negative							Positive	Negative			
≤60 years	Positive	144	1	145	84.70 (65.84–1.00)	99.54 (97.36–99.99)	93.06 (90.46–95.58)	99.31 (96.22–99.98)	89.34 (85.42–93.22)	133	2	135	96.66 (94.35–98.21)	0.9274
	Negative	26	218	244						11	243	254		
	Total	170	219	389						144	245	389		
>60 years	Positive	56	6	62	94.92 (85.85–98.94)	88.46 (76.56–95.65)	91.89 (85.17–96.23)	90.32 (80.12–96.36)	93.88 (83.13–98.72)	55	4	59	95.50 (89.80–98.52)	0.9099
	Negative	3	46	49						1	51	52		
	Total	59	52	111						56	55	111		

Calculations: Negative predictive value (NPV) = true negative/(true negative + false negative); Positive predictive value (PPV) = true positive/(true positive + false positive); Sensitivity = true positive/(true positive + false negative); Specificity = true negative/(true negative + false positive); Total coincidence rate = (true positive + true negative)/total number.

Among the 111 subjects aged >60 years, test kit results of 102 subjects were consistent with clinical diagnosis. The total coincidence rate between the test kit and clinical diagnosis was 91.89% (95% CI: 85.17–96.23), with a sensitivity of 94.92% (95% CI: 85.85–98.94), a specificity of 88.46% (95% CI: 76.56–95.65), a PPV of 90.32% (95% CI: 80.12 to 96.36) and a NPV of 93.88% (95% CI: 83.13 to 98.72). The total coincidence rate between test kit and control kit was 95.50% (95% CI: 89.80 to 98.52), and the Kappa index was 0.9099.

## Discussion

The rapid and accurate detection of SARS-CoV-2 is critical to ensure rapid and proper patient management, control outbreaks and gain a better understanding of the global epidemiology of the virus [10]. However, due to the insufficient supply of kits for detection of SARS-CoV-2, only some suspected cases are detected, resulting in incomplete data and inaccuracy in updating new cases, as well as delayed diagnosis [11]. To meet the market demand, our company has developed a new diagnostic kit (the Multiple Real-Time PCR Kit) based on RT-PCR. In the present study, we aimed to evaluate the accuracy of this kit for detection of SARS-CoV-2 compared with the kit produced by Shanghai ZJ Bio-Tech Co.

On the basis of the Diagnosis and Treatment of Pneumonitis Caused by SARS-CoV-2 (trial seventh version) guideline, diagnosis was based on clinical history, laboratory results, chest radiographic findings and nucleic acid-based assays [9]. In the present study, 242 subjects were clinically diagnosed with SARS-CoV-2 infection. Compared with clinical diagnosis, the sensitivity of the test kit was 82.64%, specificity was 98.45%, total coincidence rate was 90.80% and the Kappa index was 0.8149. Moreover, the total coincidence rate between the control kit and clinical diagnosis was 89.20%, with a sensitivity of 78.10%, a specificity of 99.61% and a Kappa index of 0.7823. These results indicated that the kit developed by Beijing Applied Biological Technologies Co. was more accurate for detecting SARS-CoV-2, with higher sensitivity, total coincidence rate and Kappa index.

Virus nucleic acid RT-PCR is the most frequently used tool for SARS-CoV-2 detection. However, previous studies have reported that an important issue with RT-PCR was the risk of eliciting false-positive and false-negative results [12–14]. A false-positive result may cause the subject to undergo unnecessary treatment, and he or she may be infected by other true-positive patients; a false-negative result could delay medical assistance for the subject and play a decisive role in the spread of pandemic infection [15]. Among the 500 subjects in our study, the test kit results of 46 subjects (false-positive: 4; false-negative: 42) were inconsistent with clinical diagnosis, whereas the control kit results of 54 subjects (false-positive: 1; false-negative: 53) were inconsistent with clinical diagnosis. One study reported sputum to be the most accurate specimen for laboratory diagnosis of SARS-CoV-2, followed by nasopharyngeal swab; oropharyngeal swab was not recommended for diagnosis [16]. In the present study, most specimens were from oropharyngeal swabs (70.3%), which may explain the false-positive and false-negative results.

We further analyzed agreement of the diagnostic results between the two kits. Compared with the control kit, the sensitivity of the test kit was 99.08%, specificity was 94.78%, total coincidence rate was 96.44% and Kappa index was 0.9259. These results indicate that the two kits were in good agreement. In addition, among the 562 specimens, test kit results of 20 specimens were inconsistent with the control kit. Through  further analysis of the inconsistent specimens, we found that the test kit results of 15 specimens were consistent with clinical diagnosis, which further proved that our kit had a greater diagnostic accuracy.

There are several limitations to the current study. First, most of the specimens in this study were from oropharyngeal swabs, which affected the accuracy of diagnosis to some extent. Second, not all tests were conducted on the same day due to workflow and personnel constraints, although all tests were completed within 72 h of specimen collection.

## Conclusion

The Multiple Real-Time PCR Kit developed by Beijing Applied Biological Technologies Co. was comparable in sensitivity, specificity and total coincidence rate to a similar detection kit for detection of SARS-CoV-2.

**Summary Points**

- The outbreak of severe acute respiratory syndrome-coronavirus 2 (SARS-CoV-2) has caused serious harm worldwide. Therefore, accurate and rapid diagnosis of SARS-CoV-2 infection is extremely important to disrupt the transmission chain.
- The new commercial Multiple Real-Time PCR Kit for detection of SARS-CoV-2 was developed by Beijing Applied Biological Technologies Co. In this study, we aimed to evaluate the accuracy of the new kit for detection of SARS-CoV-2 compared with a similar detection kit.
- The new test kit showed good performance in lowest detection limit, freeze–thaw stability, cross-reactivity and anti-interference ability.
- Compared with clinical diagnosis, sensitivity of test kit was 82.64%, specificity was 98.45%, positive predictive value was 98.04%, negative predictive value was 85.81% and the total coincidence rate was 90.80%.
- The total coincidence rate between control kit and clinical diagnosis was 89.20%, with sensitivity of 78.10%, specificity of 99.61%, positive predictive value of 99.47% and negative predictive value of 82.90%.
- The Multiple Real-Time PCR Kit developed by Beijing Applied Biological Technologies Co. was comparable to the similar detection kit for detection of SARS-CoV-2 in sensitivity, specificity and total coincidence rate.
